# Efficacy and Safety of Rho Kinase Inhibitors vs. Beta-Blockers in Primary Open-Angle Glaucoma: A Systematic Review with Meta-Analysis

**DOI:** 10.3390/jcm13061747

**Published:** 2024-03-18

**Authors:** Brenda Nana Wandji, Noélie Bacq, Adèle Ehongo

**Affiliations:** 1 Hôpital Universitaire de Bruxelles (HUB), CUB Hôpital Erasme, Service d’Ophtalmologie, Université Libre de Bruxelles (ULB), Route de Lennik 808, 1070 Bruxelles, Belgium; 2 Ecole de Santé Publique, Université Libre de Bruxelles, 1070 Brussels, Belgium

**Keywords:** Rho kinase inhibitors, beta-blockers, intraocular pressure, glaucoma, adverse effects, adults, systematic review, meta-analysis, efficacy, alternative therapy

## Abstract

**Background:** In order to support the positioning of Rho kinase inhibitors (Rhokis) in the European market for the treatment of glaucoma, scientific evidence comparing the efficacy and safety of Rhokis and beta-blockers (β-βs) in the treatment of open-angle glaucoma after 3 months was assembled through a systematic review and meta-analysis (meta-A) of randomized controlled trials (RCTs). **Methods:** Relevant articles were searched for on PubMed, EMBASE, and the Cochrane Library. Of the 251 articles found, three met all eligibility criteria. These three articles were assessed for risk of bias. Data were extracted and a random effects meta-A was performed. The studies’ methods were homogeneous but there was great heterogeneity within the data (I^2^ = 92–93%; *p* < 0.001). **Results:** All studies had low risk of bias. The meta-A showed statistically better efficacy of β-βs, resulting in an intraocular pressure (IOP) reduction mean difference of 1.73 (1.19–2.27) at 8 a.m., 0.66 (0.19–1.15) at 10 a.m. and 0.49 mmHg (0.001–0.98) at 4 p.m., compared to Rhokis. This difference is not clinically significant as intra-operator variability of IOP measurements varies from ±2 to ±3 mmHg The adverse effects of Rhokis were essentially topical, whereas β-βs mainly caused systemic side effects. **Conclusions:** This Meta-A showed that Rhokis are clinically non-inferior to beta-blockers in reducing IOP. Rhokis have a better safety profile.

## 1. Introduction

Glaucoma refers to a group of progressive optic neuropathies characterized by excavation or cupping of the optic disc, apoptotic degeneration of retinal ganglion cells, and corresponding visual field defects [1,2]. Glaucoma is the leading cause of irreversible blindness, affecting around 57.5 million people worldwide in 2015 [3,4]. With the growing number and proportion of elderly people, it is projected that 111.8 million people will have glaucoma in 2040 [3,5]. It has a prevalence of 2.6% in Europe [6]. Glaucomas can be classified into open-angle glaucomas and angle-closure glaucomas to describe the anatomic status of the anterior chamber angle. Each of these is further divided into primary or secondary types, indicating the absence or presence of other clinically identifiable ocular or systemic disorders causing the glaucoma [2].

Glaucoma is a multifactorial disease and its pathogenesis is incompletely understood. Although there are a substantial number of cases of normal pressure glaucoma [7], the main objective in the management of glaucoma is still to reduce the intraocular pressure (IOP) [8]. Several classes of drugs are used for this purpose, with prostaglandin F2α analogues and beta-blockers being the most widespread [9]. Beta-blockers have adverse cardiovascular and respiratory effects (bronchospasm, bradycardia, hypotension, arrhythmia, and reduced ventricular ejection fraction) in up to 13% of patients, and neurological effects (dizziness, hallucinations, and confusion) in three to 10% of patients [10]. These effects are even more serious in elderly patients with multiple comorbidities [11,12]. 

Over the past few years, new classes of drugs have been developed to reduce IOP, such as Rho kinase inhibitors. This therapeutic class has shown its efficacy in reducing IOP, both in monotherapy and combination therapy [13]. The added value of this group lies in their neuroprotective and vasoactive properties, such as cell survival and axon regeneration [14]. Moreover, Rho kinase inhibitors have mainly topical side effects, generally conjunctival hyperemia [15]. This new therapeutic class was approved in Japan in 2014 and in the USA in 2017 [16], but not in Europe, where studies have only been conducted, in Germany, since 2021 [17].

Studies providing high levels of scientific evidence would support arguments for the introduction of Rho kinase inhibitors to the European market. This would diversify the therapeutic options available for glaucoma patients, because Rho kinase inhibitors have also been shown to further reduce IOP in patients previously treated with drugs from other therapeutic classes [18]. Moreover, it is beneficial to have a wide therapeutic choice in case of side effects, intolerance, or allergies with other therapeutic classes, particularly for elderly patients and/or those with co-morbidities, in whom beta-blockers can be responsible for life threatening adverse effects [19,20,21].

So far, no meta-analysis of randomized controlled trials comparing Rho kinase inhibitors and beta-blockers in open-angle glaucoma is available in the published literature. Therefore, the main objective of this review was to assemble scientific evidence related to the efficacy and safety of Rho kinase inhibitors compared to beta-blockers in reducing IOP in patients with primary open-angle glaucoma.

## 2. Materials and Methods

A literature search was conducted using online databases (PubMed, EMBASE, Google scholar, and the Cochrane Library) for studies published from 2001 to 1 July 2023. Specific search strategies were developed for each database. This review was performed in accordance with the PRISMA (Preferred Reporting Items for Systematic Reviews and Meta-Analyses) guidelines [22].

Data were extracted from 1 May to 1 July 2023. The following search terms were used: “open angle glaucoma” for population; “Rho Kinase inhibitor” for intervention; “beta-blockers” for comparison; and “intraocular pressure” for outcome. Synonyms of search terms were combined using “OR,” whereas population, intervention, comparison and outcome were combined by “AND.”

An example of the research strategy for PubMed was: (“Rho associated Kinases”[MeSH Terms] OR (“Rho associated”[All Fields] AND “Kinases”[All Fields]) OR “Rho associated Kinases”[All Fields] OR (“Rho”[All Fields] AND “Kinase”[All Fields]) OR “Rho Kinase”[All Fields]) AND (“antagonists and inhibitors”[MeSH Subheading] OR (“antagonists”[All Fields] AND “inhibitors”[All Fields]) OR “antagonists and inhibitors”[All Fields] OR “inhibitors”[All Fields] OR “inhibitor”[All Fields] OR “inhibitor s”[All Fields]) AND (“beta-blocker”[All Fields] OR “beta-blockers”[All Fields] OR “betablocking”[All Fields]) AND (“glaucoma, open angle”[MeSH Terms] OR (“glaucoma”[All Fields] AND “open angle”[All Fields]) OR “open-angle glaucoma”[All Fields] OR (“open”[All Fields] AND “angle”[All Fields] AND “glaucoma”[All Fields]) OR “open angle glaucoma”[All Fields]).

The selection was based on the acronym “PICOS”:

- Patients: performed on adults, 18 years old or more with a primary open-angle glaucoma diagnosis based on gonioscopy, OCT, and visual field defects with or without ocular hypertension.
- Intervention: Rho kinase inhibitors treatment with no additional concomitant therapy for at least 3 months.
- Comparison: beta-blockers treatment with no additional concomitant therapy for at least 3 months.
- Outcome: the reduction of IOP after a 3-month treatment was assessed by subtracting the IOP recorded at 3 months after medication from the IOP at baseline (ΔIOP = IOP at 3 months − IOP at baseline). A negative value of ΔIOP implies that the study drug is effective in lowering IOP. From each study, the mean difference of the IOP reduction was extracted at various times because IOP values can fluctuate through the day. The occurrences of systemic and topical adverse effects in both groups were also assessed.

Study design: randomized controlled trials (RCTs) published in English or French between 2001 (the publication year of the first studies on Rho kinase inhibitors for the treatment of glaucoma) and 2023 were included. The following were excluded:

- Case reports, systematic reviews, books, editorials, opinions, grey literature.
- Studies on types of glaucoma other than primary open-angle glaucoma.
- Studies in which additional therapies or surgical procedures for glaucoma management were also performed.
- Studies which did not specify the required data (IOP difference before and after treatment).

Titles and abstracts were assessed according to the eligibility criteria, then duplicates were excluded using Rayyan software (https://www.rayyan.ai/, Rayyan Systems Inc., Cambridge, MA, USA). The full texts of studies whose titles/abstracts contained insufficient information for a decision were also assessed. The studies that met the eligibility criteria were included in this systematic review and meta-analysis. A complementary search of the reference lists of studies included in this systematic review was performed manually.

Two researchers (BN and NB) extracted data from the included articles. The following data were extracted: authors/year of publication, study design, number of participants, characteristics of the samples (sex and participants’ age), the name of the beta-blocker and Rho kinase inhibitor used, mean values and standard deviation for the IOP, the follow-up duration, and the proportions of local and general adverse effects. Disagreements between the two researchers were resolved with the aid of a third researcher (AE).

The risk analysis for bias was performed using a tool called ROB-2 [23]. The following fields are assessed by this tool: (1) bias arising from the randomization process, (2) deviations from intended interventions, (3) missing outcome data, (4) measurement of outcomes, and (5) selection of the reported results. Each item contains several questions, and the response to each question is used to classify the study in terms of low/moderate/high risk of bias. The total score of the item is reported according to the category in which most responses fall. 

Two researchers (BN and NB) conducted the analysis independently, and the results were compared until a consensus was reached.

Data were analyzed using Stata version 17 (StataCorp. 2021. Stata Statistical Software: Release 17. College Station, TX: StataCorp LLC, College Station, TX, 77845 USA). A meta-analysis with random effects model was used. The effect size was estimated using Cohen’s d (since the sample for each study was >10) with the corresponding 95% confidence interval. We calculated the I^2^ statistics with the following classification of degree of heterogeneity: <25% (low heterogeneity), 25–50% (moderate heterogeneity), >50% (high heterogeneity). We assessed H^2^ as the ratio of the variance of the estimated overall effect size from a random-effects meta-analysis compared to the variance from a fixed-effects meta-analysis. There was perfect homogeneity across studies when H^2^ equaled 1, and the greater the value of H^2^, the greater was the heterogeneity. *p* values of less than 0.05 were considered statistically significant.

## 3. Results

### 3.1. Study Selection

**Figure 1:**
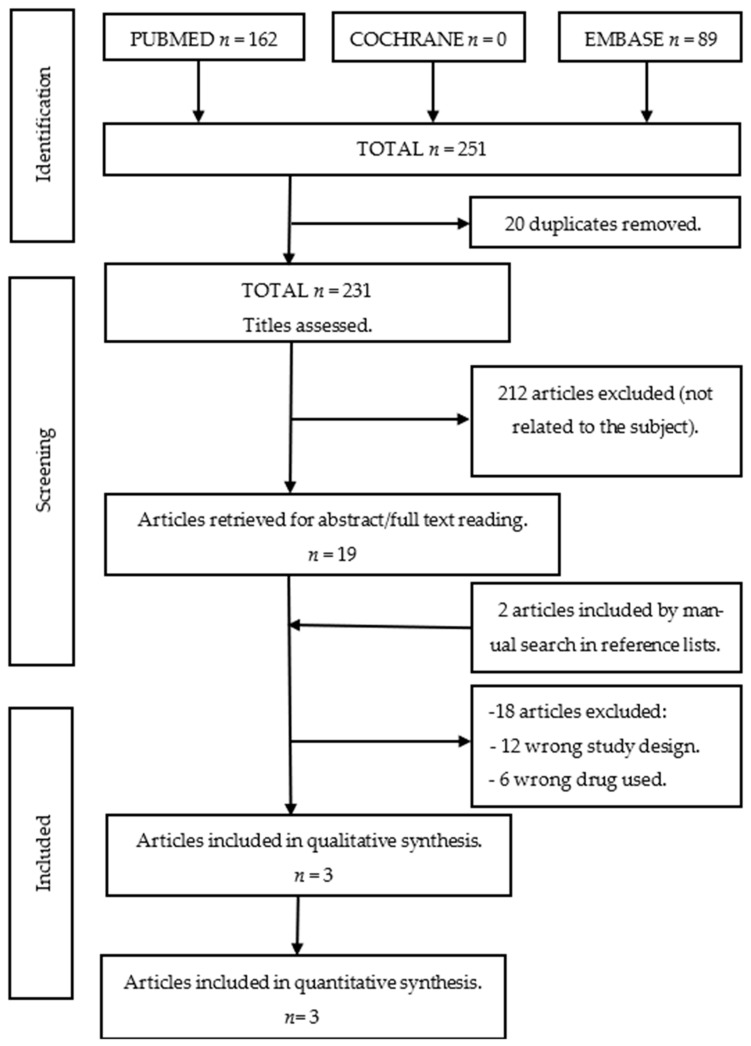
Flowchart of study selection according to PRISMA guidelines.

The electronic searches identified 251 studies, of which 20 duplicates were removed. Three articles were included in this systematic review and meta-analysis. The article selection process is presented in Figure 1.

### 3.2. Characteristics of Articles

#### 3.2.1. Description of the Articles

Three studies of an intervention named “ROCKET” (Rho kinase-Elevated IOP Treatment) were found. They were phase III RCTs, all conducted in the US by Serle et al. in 2018 (ROCKET 1) [24], Kahook et al. in 2019 (ROCKET 2) [25], and Khouri et al. (ROCKET 4) in 2019 [26]. These studies compared the efficacy of IOP reduction in open-angle glaucomatous patients treated with twice-daily administration of a beta-blocker (timolol 0.5%) and a daily administration of a Rho kinase inhibitor (netarsudil 0.02%). IOP was measured on three occasions: at 8 a.m., 10 a.m. and 4 p.m. To ensure blinding control, patients belonging to the group on once daily netarsudil were given a placebo in the morning and an active drug in the evening. The ROCKET 2 study also compared efficacy after twice-daily administration of netarsudil. These data were not included in the present meta-analysis, as only the ROCKET 2 study obtained those results. As the follow-up period ranged from 3 to 12 months in the ROCKET studies, for the sake of uniformity this meta-analysis was performed on measurements obtained after a 3-month treatment period.

#### 3.2.2. Demographic Characteristics

The mean age of participants in the included studies ranged between 63 ± 11.8 and 65 ± 11.5 years. The sample sizes ranged from 411 to 756, with a female predominance in each treatment group (59–66% women). Randomization was good, as the groups were comparable in terms of sex and age.

#### 3.2.3. Intraocular Pressure

The articles examined individually concluded that netarsudil was not significantly inferior (non-inferior) to timolol after a 3-month treatment regime. 

Appendix A, Table A1 and Table A2 show additional information regarding the demographic characteristics and results of the articles included in this systematic review and meta-analysis. 

#### 3.2.4. Risk of Bias

Table 1 shows the results of the evaluation of the risk of bias of the included articles using the ROB-2 tools. Overall, the risk of bias was low in the included studies except for ROCKET 2, which had a high risk of bias in terms of missing data.

### 3.3. Assessment of Efficacy

#### 3.3.1. Comparison of IOP Reduction at 8 a.m.

**Figure 2:**
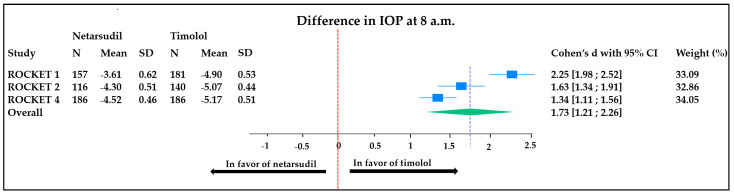
Forest plot representing the difference in IOP (measured at 8 a.m.) between patients treated with timolol vs. netarsudil. Heterogeneity: T^2^ = 0.20, I^2^ = 91.96%, H^2^ = 12.43; test of θi = θj: Q (2) = 25.71, *p* < 0.001; test of θ = 0: z = 6.44, *p* < 0.001. ROCKET: Rho kinase-Elevated IOP Treatment; SD: standard deviation; CI: confidence interval; IOP: intra ocular pressure.

The overall mean difference in IOP reduction at 8 a.m. was 1.73 mmHg (95% CI, 1.19 to 2.27). This means that the timolol reduces the IOP of 1.73 mmHg significantly more than netarsudil after a 3-month therapy regime. A significantly high heterogeneity between studies was found (I^2^ = 92.2%) (Figure 2); the weight of each study was comparable. The sensitivity analysis did not reveal any significant changes in the overall effect size when individual studies were excluded, suggesting that the observed heterogeneity may not be solely driven by a specific study (Figure 2).

#### 3.3.2. Comparison of IOP Reduction at 10 a.m.

**Figure 3:**
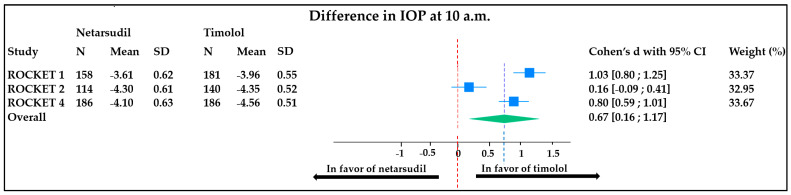
Forest plot representing the difference in IOP (measured at 10 a.m.) between patients treated with timolol vs. netarsudil. Heterogeneity: T^2^ = 0.19, I^2^ = 93.23%, H^2^ = 14.77; test of θi = θj: Q (2) = 27.18, *p* < 0.001; test of θ = 0: z = 2.58, *p* = 0.01. ROCKET: Rho kinase-Elevated IOP Treatment; SD: standard deviation; CI: confidence interval; IOP: intra ocular pressure.

At 10 a.m., there was an overall mean difference in IOP reduction of 0.67 mmHg (95% CI, 0.16 to 1.17). This means that timolol reduces the IOP of 0.67 mmHg significantly more than netarsudil after a 3-month therapy regime. The weight of each study was comparable. There was a significantly high heterogeneity between studies (I^2^ = 92%) (Figure 3). 

#### 3.3.3. Comparison of IOP Reduction at 4 p.m.

**Figure 4:**
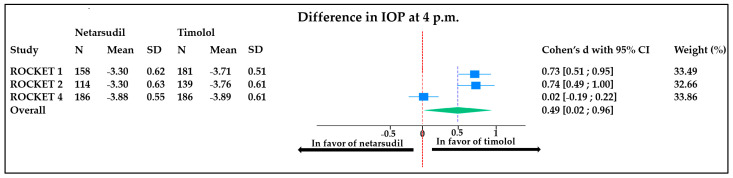
Forest plot representing the difference in IOP (measured at 4 p.m.) between patients treated with timolol vs. netarsudil. Heterogeneity: T^2^ = 0.16, I^2^ = 92.41%, H^2^= 13.17; test of θi = θj: Q (2) = 28.52, *p* < 0.001; test of θ = 0: z = 2.04, *p* = 0.04. ROCKET: Rho kinase-Elevated IOP Treatment; SD: standard deviation; CI: confidence interval; IOP: intra ocular pressure.

At 4 p.m., there was an overall mean difference in IOP reduction of 0.49 mmHg (95% CI, 0.02 to 0.96). This means that the timolol reduces the IOP of 0.49 mmHg significantly more than netarsudil after 3 months of therapy. The weight of each study was comparable, although a significantly high heterogeneity between studies was found (I^2^ = 93%) (Figure 4). 

### 3.4. Assessment of Safety

#### 3.4.1. Topical Adverse Effects

Topical side effects were significantly more common in the netarsudil-treated group in all studies. The most common side effects were conjunctival hyperemia, cornea verticillata, and subconjunctival hemorrhage (Table 2).

**Figure 5:**
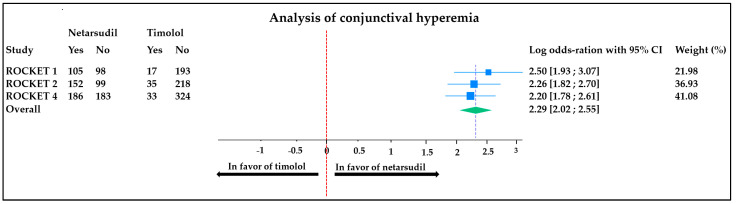
Comparison of conjunctival hyperemia occurrence in patients treated with netarsudil vs. timolol. Heterogeneity: T^2^ = 0.00, I^2^ = 0.00%, H^2^ = 1.00; test of θi = θj: Q (2) = 0.72, *p* = 0.70; test of θ = 0: z = 16.85, *p* < 0.001. ROCKET: Rho kinase-Elevated IOP Treatment; CI: confidence interval.

**Figure 6:**
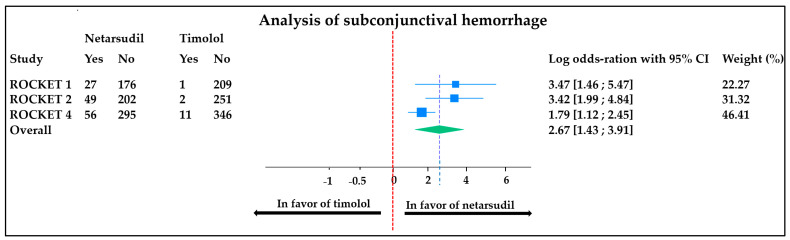
Comparison of subconjunctival hemorrhage occurrence in patients treated with netarsudil vs. timolol. Heterogeneity: T^2^ = 0.75, I^2^ = 63.15%, H^2^= 2.71; test of θi = θj: Q (2) = 5.81, *p* = 0.05; test of θ = 0: 4.23, *p* < 0.001. ROCKET: Rh kinase-Elevated IOP Treatment; CI: confidence interval.

The meta-analyses comparing conjunctival hyperemia and subconjunctival hemorrhage occurrence between groups (the side effects for which all data were available) are displayed in Figure 5 and Figure 6, respectively. 

Conjunctival hyperemia: the meta-analysis carried out on conjunctival hyperemia revealed a minor heterogeneity (I^2^ = 0%) between studies, with an overall odds ratio (OR) of 2.29 [2.02;2.55]. This means that there was significantly more (2.29 times more) conjunctival hyperemia in patients treated with netarsudil than in those treated with timolol (Figure 5).

Subconjunctival hemorrhage: the meta-analysis carried out on subconjunctival hemorrhage revealed high heterogeneity (I^2^ = 63.15%) between studies, with an overall OR of 2.67 [1.43;3.91]. This means there were significantly more (2.67 times more) subconjunctival hemorrhages in patients treated with netarsudil than in patients treated with timolol (Figure 6).

#### 3.4.2. Systemic Adverse Effects

In the ROCKET 1 study, the systemic side effects were not listed. In the ROCKET 2 study, there was a significant reduction in heart rate of −2.1 beat/min with timolol therapy, while the heart rate was not changed significantly in the group receiving netarsudil therapy. The exact data were not included in the study. There was a significantly higher proportion of adverse musculoskeletal effects in patients treated with timolol (*p* = 0.01) (Table 3).

In the ROCKET 4 study, the systemic side effects were not listed according to the body systems affected as in ROCKET 2, except for heart rate. There was a significant reduction in heart rate of −2 beat/min with timolol therapy, meanwhile the heart rate was not changed significantly in the group receiving netarsudil therapy (Table 3). Moreover, the overall systemic side effects were more frequent in the timolol group *n* = 91 (25.5%) than in the netarsudil group *n* = 82 (23.4%). This difference was not statistically significant (*p =* 0.9).

A meta-analysis of systemic side effects could not be performed because the data needed for the calculation were not provided by the studies.

## 4. Discussion

Efficacy: In the present study, the efficacy and safety of Rho kinase inhibitors versus beta-blockers in reducing IOP in glaucomatous patients were compared through a meta-analysis. The studies included were RCTs with a low overall risk of bias. Individually, the studies concluded that there was a non-inferiority of netarsudil compared to timolol in reducing IOP. But, once the effects were combined, a statistically significant superiority of timolol was observed. This could be because the authors chose a non-inferiority cut-off point of 10%, i.e., if the mean difference in IOP reduction between the two molecules was less than 10%, then they concluded that the efficacy was the same. 

Furthermore, these studies involved only a single Rho kinase inhibitor, netarsudil. Yet, other Rho kinase inhibitors such as ripasudil have been proven to be genuinely effective in reducing IOP [27]. There was no study comparing the efficacy of ripasudil with a beta-blocker used alone.

Finally, the difference in IOP reduction between the two treatments, ranging from 0.49 to 1.73 mmHg in favor of timolol, although statistically significant, is not clinically relevant, as intra-operator variability in IOP measurements with an applanation tonometer varies from ±2 to ±3 mmHg [28].

The non-inferiority of Rho kinase inhibitors compared to beta-blockers is a key point for positioning Rho kinase inhibitors in the therapeutic arsenal against glaucoma.

Safety: Topical side effects were significantly more common in the netarsudil-treated groups. These included conjunctival hyperemia, which was the most common, followed by subconjunctival hemorrhage and cornea verticillata. Nevertheless, they were minor and were consistent with those reported in other Rho kinase studies [15,17]. Conversely, systemic side effects were significantly more frequent in patients treated with timolol, notably bradycardia, and musculoskeletal and gastrointestinal disorders. It is well known that beta-blocker eye drops can induce serious complications which can be life threatening, especially in the elderly or those with pre-existing comorbidities [19,20,21]. Overall, the better systemic safety profile of Rho kinase inhibitors is critical, as the choice of which IOP-reducing medication to use is dictated by the compatibility between the patient’s comorbidity state and the sides effects of the target molecule.

Clinical setting: This meta-analysis shows that netarsudil is not inferior to timolol and that, in addition, it has a better systemic safety profile. Netarsudil appears, therefore, to be a good option for first-line treatment. It is also a valuable alternative when the patient does not tolerate other treatments, or when beta-blockers are contra-indicated.

Guidelines for the treatment of glaucoma recommend combining molecules with different mechanisms of actions for greater effectiveness [29]. While the mechanism of action of beta-blockers is through the reduction of aqueous humor production, and that of prostaglandin analogs is through the enhancement of the uveoscleral pathway, Rho kinase inhibitors provide an alternative mechanism. They reduce IOP by improving the conventional aqueous outflow pathway via cytoskeletal redistribution and changes in cell–cell interactions in the endothelial cells of the trabecular meshwork and Schlemm’s canal. This alternative mechanism of IOP reduction, compared to other classes of IOP medications, therefore offers the potential for additive IOP reduction as a combination treatment [30].

Rho kinase inhibitors can thus be combined with other medications for greater effectiveness, especially when a high IOP reduction is expected. In this regard, Tanihara et al., in 2015, found significant additive IOP reduction ranging from 0.9 mmHg (95% CI, 0.4–1.3 mmHg; *p* < 0.001) to 1.6 mmHg (95% CI, 1.1–2.1 mmHg; *p* < 0.001) with combined therapy of ripasudil with timolol, compared to timolol alone [30]. Likewise, Lee et al., in 2022, found a reduction of −2.41 mmHg (95% confidence interval [CI], −2.95 to −1.87) with combined therapy of netarsudil with latanoprost, compared to −1.77 mmHg (95% CI, −2.31 to −1.87) with latanoprost alone [31]. 

**Figure A1:**
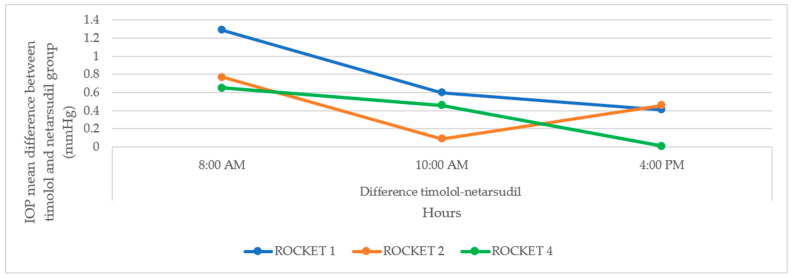
Hourly variation in mean IOP difference between netarsudil and timolol groups. ROCKET: Rho kinase-Elevated IOP Treatment; IOP: intra ocular pressure.

It is interesting to note that the difference in efficacy between netarsudil and timolol decreased from the morning (10 a.m. measurements) to the evening (4 p.m. measurements). Starting at 8 a.m., the mean difference in IOP between the two groups varied from 1.29 mmHg to 0.65 mmHg, depending on the study. As the day progressed, this difference narrowed between the two groups, to the point where at 4 p.m., in the Rocket 4 study, the difference was almost zero (0.01 mmHg) (Appendix A, Figure A1). This suggests that the IOP lowering effect of netarsudil may be more constant than that of timolol. This is consistent with the longer half-life of netarsudil previously reported [32]. Therefore, netarsudil has the potential to better protect visual function in the long term As one risk factor of glaucoma progression is IOP fluctuations, this warrants further studies.

Furthermore, in the included studies, the IOP measurements were taken during the time slots when timolol was at its peak activity, while netarsudil’s peak activity had passed. The peak activity of timolol occurs 2 h after instillation [33], and that of netarsudil 4 to 8 h after instillation [34]. Therefore, based on a single evening dose of netarsudil and a morning dose of timolol, IOP measurements taken between 8 a.m. and 4 p.m. would fall within the period of peak activity of timolol and low activity of netarsudil. 

Moreover, as out-of-office hours were not evaluated in these studies, it is possible that during these hours netarsudil is more potent than timolol, because timolol is less effective at night [35], while netarsudil would be at its highest activity after instillation in the evening.

Finally, the authors did not perform IOP measurements after 4 p.m. It would be interesting to take IOP measurements just before the evening dose, as the persistent efficacy of a drop is protective against non-compliance in real life.

Limitations and recommendations: The limitations encountered in this study lie mainly in the paucity of clinical trials on the efficacy of Rho kinase inhibitors compared to beta-blockers. Second, the selected studies were essentially all carried out in the USA by the same team. The results could therefore not be extrapolated to Europe. Hence the need for pilot studies on European populations. Third, the selected studies did not discuss the long-term safety and efficacy of netarsudil in the management of glaucoma, which is a chronic condition. This may be related to the fact that Rho kinase inhibitors have only recently been used in the treatment of glaucoma. Long-term follow-up studies would therefore be worthwhile. Further studies analyzing evening measurements before instillation of the next dose of drops would provide information on whether Rho kinase inhibitors have a sustained 24 h IOP-lowering effect compared to beta-blockers. Also, assessing long-term functional and structural evolution would help determine whether Rho kinase inhibitors better reduce the progression of glaucoma. Finally, there was an absence of useful data, such as means and standard deviations, in the results of the selected studies. Attempts to contact the authors to obtain further information were not successful. It is therefore recommended that researchers provide readers (in their appendices at least) with as much information as possible about their study, especially in the case of pilot research.

## 5. Conclusions

In the current study, it was shown that Rho kinase inhibitors, specifically netarsudil, are clinically non-inferior to beta-blockers in reducing IOP. Moreover, Rho kinase inhibitors offer added benefits in the management of glaucoma as alternatives to other drug classes, mainly beta-blockers, because their side effects are essentially localized and harmless. It is suggested to carry out more RCTs on Rho kinase inhibitors, with a longer follow-up period and including European populations, and assessing Rho kinase inhibitors other than netarsudil. Finally, it is suggested to measure the IOP-lowering effect of Rho kinase inhibitors shortly before and after the evening dose administration.

## Figures and Tables

**Table 1:** Risk of bias assessment of the included studies according to the ROB-2 tool.

Study	Randomization Process	Deviations from Interventions	Missing Data	Outcome Measurement	Reported Results Selection	Overall Bias
ROCKET 1	Low	Low	Low	Low	Low	Low
ROCKET 2	Low	Low	High	Low	Low	Low
ROCKET 4	Low	Low	Low	Low	Low	Low

ROCKET: Rho kinase-Elevated IOP Treatment.

**Table 2:** Main local adverse effects in patients treated with netarsudil vs. timolol in each study.

Local Adverse Effects												
	Conjunctival Hyperemia			Cornea Verticillata			Subconjunctival Hemorrhage			Blurred Vision		
	Netarsudil	Timolol		Netarsudil	Timolol		Netarsudil	Timolol		Netarsudil	Timolol	
Study	*n* (%)	*n* (%)	*p*	*n* (%)	*n* (%)	*p*	*n* (%)	*n* (%)	*p*	*n* (%)	*n* (%)	*p*
ROCKET 1	105 (51.7)	17 (8.1)	<0.001	11 (5.4)	NA		27 (13.3)	1 (0.5)	<0.001	NA	NA	
ROCKET 2	152 (60.5)	35 (13.9)	<0.001	64 (25.5)	2 (0.8)	<0.001	49 (19.5)	2 (0.8)	<0.001	27 (10.7)	7 (2.8)	<0.001
ROCKET 4	168 (47.9)	33 (9.2)	<0.001	86 (24.5)	0 (0)	<0.001	56 (16.0)	11 (3.1)	<0.001	22 (6.3)	4 (1.1)	0.05

NA: Not available. ROCKET: Rho kinase-Elevated IOP Treatment.

**Table 3:** Main systemic adverse effects in patients treated with netarsudil vs. timolol in each study.

Systemic Adverse Effects													
	Heart Rate (beat/min)				Respiratory/Thoracic			Musculoskeletal			Gastrointestinal		
	Netarsudil		Timolol		Netarsudil	Timolol		Netarsudil	Timolol		Netarsudil	Timolol	
Study	MD ± SD	*p*	MD ± SD	*p*	*n* (%)	*n* (%)	*p*	*n* (%)	*n* (%)	*p*	*n* (%)	*n* (%)	*p*
ROCKET 1	NA	NA	NA	NA	NA	NA	NA	NA	NA	NA	NA	NA	NA
ROCKET 2	NA	NS	−2.1 ± 0.6	*p* < 0.001	10 (3.9)	14 (5.6)	0.5	3 (1.2)	17 (6.8)	0.01	2 (0.8)	9 (3.6)	0.06
ROCKET 4	0.8 ± 1.0	NS	−2 ± 1.0	*p* < 0.001	NA	NA	NA	NA	NA	NA	NA	NA	NA

NA: Not available; NS: non-significant: the exact *p* value was not specified in the study; MD = mean difference; SD: standard deviation; CI: confidence interval; ROCKET: Rho kinase-Elevated IOP Treatment.

## Data Availability

Data sharing is not applicable as no new data was generated and the article describes entirely theoretical research.

## Appendix A

**Table A1:** Characteristics of studies included in the meta-analysis.

Study	Authors Year	Sample Size (*n*)	Inclusion Criteria		Follow Up. Duration
			Age (Years) Mean ± SD	Diagnosis	
ROCKET 1	SERLE et al., 2018 [24]	411	65.1 ± 11.5	OAG or OHT >17 and <27 mmHg	Week 2 and 6, Month 3
	Netarsudil q.d	202	65.2 ± 11.3		
	Timolol b.i.d	209	NA		
ROCKET 2	KAHOOK et al. 2019 [25]	756	65.3 ± 11.48	OAG or OHT IOP < 25 mmHg	Week 2 and 6 Month 3, 6, 9 and 12
	Netarsudil q.d	251	NA		
	Timolol b.i.d	251	63 ± 11.81		
	Netarsudil b.i.d *	254	64.1 ± 12.46	OAG or OHT IOP < 25 mmHg	Week 2 and 6 Month 3, 6, 9 and 12
ROCKET 4	KHOURI et al. 2019 [26]	708	NA	OAG or OHT IOP > 20 and <30 mmHg	Week 2 and 6, Month 3 and 6
	Netarsudil q.d	351	64.1 ± 11.6		
	Timolol b.i.d	357	64.5 ± 11.0		

All studies compared the efficacy of netarsudil 0.02% once-daily with timolol 0.5% twice-daily. Abbreviations: q.d: (quaque die) = once a day; b.i.d: (bis in die) = two times a day; ROCKET: Rho kinase-Elevated IOP Treatment; SD: standard deviation; OAG: Open Angle Glaucoma; OHT: Ocular Hypertension; IOP: Intra Ocular Pressure. NA = not available. * Data non included in the meta-analysis.

**Table A2:** Mean difference of IOP after 3 month-treatment in each group.

	8 a.m.				10 a.m.				4 p.m.			
	Netarsudil Group		Timolol Group		Netarsudil Group		Timolol Group		Netarsudil Group		Timolol Group	
Study	Mean	SD	Mean	SD	Mean	SD	Mean	SD	Mean	SD	Mean	SD
ROCKET 1	−3.61	0.62	−4.9	0.51	−3.36	0.62	−3.96	0.55	−3.3	0.62	–3.71	0.51
ROCKET 2	−4.3	0.55	−5.07	0.42	−4.26	0.61	−4.35	0.52	−3.3	0.63	−3.76	0.61
ROCKET 4	−4.52	41	−5.17	0.55	−4.1	0.63	−4.56	0.51	−3.88	0.55	−3.89	0.61

ROCKET: Rho kinase-Elevated IOP Treatment; SD: Standard Deviation; IOP: intra ocular pressure.
